# Targeting a microbiota *Wolbachian* aminoacyl-tRNA synthetase to block its pathogenic host

**DOI:** 10.1126/sciadv.ado1453

**Published:** 2024-07-10

**Authors:** Guillaume Hoffmann, Maria Lukarska, Rachel H. Clare, Ellen K.G. Masters, Kelly L. Johnston, Louise Ford, Joseph D. Turner, Steve A. Ward, Mark J. Taylor, Malene Ringkjøbing Jensen, Andrés Palencia

**Affiliations:** ^1^ Institute for Advanced Biosciences (IAB), Structural Biology of Novel Drug Targets in Human Diseases, INSERM U1209, CNRS UMR 5309, Université Grenoble-Alpes, Grenoble 38000, France.; ^2^ Centre for Drugs and Diagnostics, Department of Tropical Disease Biology, Liverpool School of Tropical Medicine, Liverpool L3 5QA, UK.; ^3^ Université Grenoble Alpes, CEA, CNRS, IBS, Grenoble, France.

## Abstract

The interplay between humans and their microbiome is crucial for various physiological processes, including nutrient absorption, immune defense, and maintaining homeostasis. Microbiome alterations can directly contribute to diseases or heighten their likelihood. This relationship extends beyond humans; microbiota play vital roles in other organisms, including eukaryotic pathogens causing severe diseases. Notably, *Wolbachia*, a bacterial microbiota, is essential for parasitic worms responsible for lymphatic filariasis and onchocerciasis, devastating human illnesses. Given the lack of rapid cures for these infections and the limitations of current treatments, new drugs are imperative. Here, we disrupt *Wolbachia*’s symbiosis with pathogens using boron-based compounds targeting an unprecedented *Wolbachia* enzyme, leucyl-tRNA synthetase (LeuRS), effectively inhibiting its growth. Through a compound demonstrating anti-*Wolbachia* efficacy in infected cells, we use biophysical experiments and x-ray crystallography to elucidate the mechanism behind *Wolbachia* LeuRS inhibition. We reveal that these compounds form adenosine-based adducts inhibiting protein synthesis. Overall, our study underscores the potential of disrupting key microbiota to control infections.

## Teaser

An approach to control infection by disrupting pathogen's microbiota is presented.

## INTRODUCTION

**Fig. 1.**
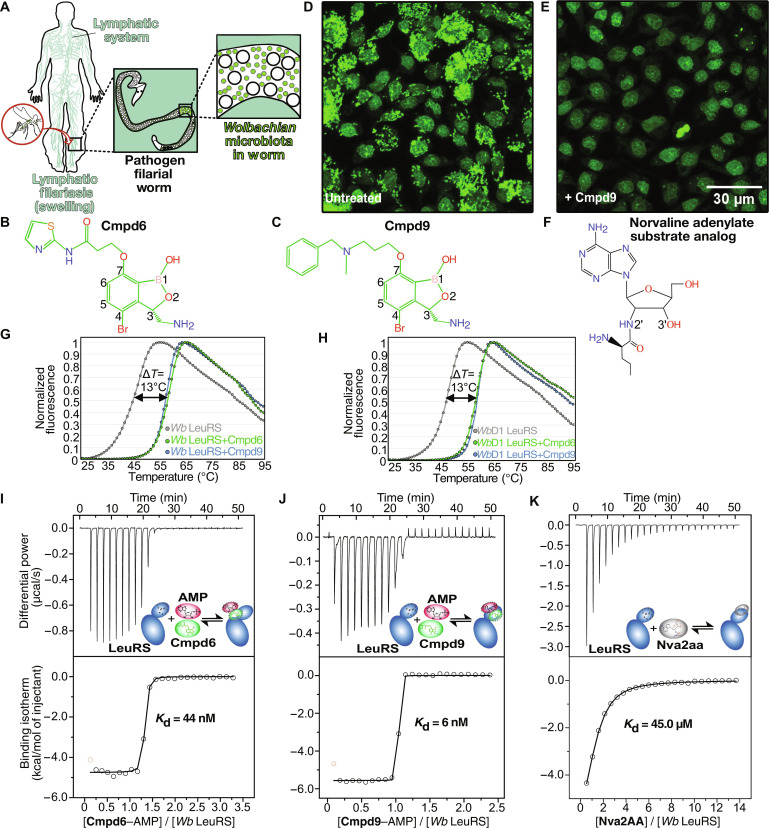
Benzoxaborole compounds target microbiota *Wolbachian* LeuRS. (**A**) Transmitted by mosquito bites, filarial worm pathogens can reach the lymphatic nodes where they cause severe chronic pain and swelling of tissues. (**B**, **C**, and **F**) Chemical structures of **Cmpd6**, **Cmpd9**, and editing substrate analog of norvaline (Nva), respectively. (**D**) C6/36 insect cells (*A. albopictus*) stably infected with *W. pipientis* (wAlbB) were analyzed by fluorescence and high-content image analysis. Single-cell image analysis shows the nucleus (fluorescent large shapes) in the center surrounded by colonizing *Wolbachia* (small fluorescent dots) in the cytoplasm. (**E**) Reduction of *Wolbachia* load in host cells treated with **Cmpd9** at 9 μM for 7 days. (**G** and **H**) Thermal-shift assays showing the stabilization of *Wolbachia* LeuRS proteins in the presence of the inhibitors **Cmpd6** and **Cmpd9**. The experiments were performed in triplicates for both the wild-type *Wolbachia* LeuRS and the construct *Wolbachia* LeuRS D1 in the presence of AMP. (**I**, to **K**) ITC binding experiments showed one order of magnitude higher affinity of **Cmpd6/9** in the presence of AMP compared to norvaline posttransfer analog (physiological editing substrate of LeuRS). DSF and ITC experiments were performed in biological and technical triplicates; *N* = 3.

Understanding the relationship between microbiota and their host species is at the forefront of medical research. There is growing evidence indicating that the interplay between microbiota and their host mammals is crucial for many cellular processes such as the absorption of food nutrients, immune defense, and homeostasis. Alterations of the population and number of microbiota species, the so-called dysbiosis, have been directly linked to the development of human diseases (*1*, *2*). As in humans, bacteria are also crucial for many other eukaryotic species, including pathogens causing major human diseases. However, specific targeting of microbiota species in strong symbiosis with their host pathogens remains an underexploited approach to control infections in humans. A remarkable example of strong symbiosis in pathogens is *Wolbachia*, a bacterium discovered nearly a century ago (*3*) that is considered one of the most successful organisms in the biosphere because of its wide colonization among arthropods and nematodes (*4*). This is due to the strong interdependence between *Wolbachia* and its hosts on successful reproduction and other symbiotic processes (*5*–*7*). Moreover, *Wolbachia* infects mosquito species that are vectors of disease to humans, and it establishes reproductive parasitism (*4*). *Wolbachia* is also an obligate intracellular bacterium vital for the survival and fertility of the parasitic worms causing lymphatic filariasis (LF) and onchocerciasis (Fig. 1A) (*8*), both devastating diseases that infect 117 million people, mostly in tropical regions. There is no rapid cure for these infections, and the current standards of care, ivermectin (for onchocerciasis) or in combination with albendazole and diethylcarbamazine (for LF), not only are contraindicated for children or women during early pregnancy, but also involve very long treatments that lack efficacy against adult worm parasites (*8*). Thus, there is an urgent need for faster and more effective drugs to cure these diseases, which could target either the pathogenic worms or, given their strong endosymbiotic relationships, their *Wolbachia* microbiota (*8*). The therapeutic benefits of inducing dysbiosis by targeting *Wolbachia* are supported by the fact that the antibiotics rifampin, tetracycline, and doxycycline can arrest worm development in preclinical models of filariasis (*9*–*14*) and by the fact that mosquito populations transmitting diseases can be controlled by targeting their *Wolbachia*n microbiota, leading to incompatible insect reproduction (*15*–*17*). However, one of the main challenges in the development of anti-filarial drugs against *Wolbachia* is the identification of novel drug targets of this microbiota because *Wolbachia* is an obligate intracellular bacterium. This severely thwarts *Wolbachia* genetic manipulation outside its hosts, making the search for essential genes by traditional genetic approaches very difficult, and the validation of the putative drug targets and the evaluation of the resistance mechanisms very challenging (*4*, *5*).

Here, we report the discovery of potent boron-based compounds that inhibit an unprecedented target of *Wolbachia*, leucyl-tRNA synthetase (LeuRS), and demonstrate that inhibition of this microbiota target is an efficient approach to arrest the growth of its host. We combine cell-based experiments showing compound efficacy in a *Wolbachia*-infection model with in vitro experiments by nuclear magnetic resonance (NMR), x-ray crystallography, and other biophysical methods to reveal the molecular basis of the *Wolbachia* LeuRS inhibition mechanism. Our work shows how targeting the LeuRS protein of pathogen microbiota is a valid approach to control infection, a strategy with potential applications to other human pathogens.

## RESULTS

### Identification of benzoxaboroles with activity against *Wolbachia* LeuRS

To identify active compounds against *Wolbachia*, we carried out a high-throughput screening assay by testing a library of 202 benzoxaborole compounds in a host-cell model of *Wolbachia* infection (*5*, *18*), which yielded an 18% hit rate. The mosquito-derived cell line C6/36 stably infected with *Wolbachia pipientis* was treated with each benzoxaborole compound at doses ranging from 10 nM to 5 μM during a short period (7 days) and was used to calculate the half-maximum effective concentration (EC_50_). The screening in cells was combined with activity experiments (aminoacylation) performed with recombinant full-length *Wolbachia* LeuRS protein, which were used to calculate the half-maximum inhibitory concentration (IC_50_) for the most interesting compounds identified in the cell screening. Thus, this yielded compounds with both good anti-*Wolbachian* in vitro activity and in vivo efficacy, with representative compounds shown in table S1. **Cmpd6** and **Cmpd9** (Fig. 1, B and C) were the most interesting compounds with IC_50_ values in the low nanomolar range (table S1). Moreover, the potent in vitro activity of **Cmpd9** translated in very good *in cellulo* efficacy (EC_50_ = 90 nM) in the *Wolbachia* infection model (Fig. 1, D and E, and table S1). This may be due to the formation of a very stable inhibition complex by **Cmpd9**, as judged by the higher half-life (also named reactivation rate) of **Cmpd9** (*T*_1/2_ = 226 min) compared to other compounds (table S1).

### Inhibition of the *Wolbachia* LeuRS editing site by Cmpd6 and Cmpd9

Next, we sought to identify the target and investigate the inhibition mechanism of these compounds. Previous studies have shown that 3-amino-methyl benzoxaboroles can inhibit the editing site of bacterial LeuRS and form adenosine-based covalent adducts with adenosine monophosphate (AMP), adenosine triphosphate (ATP), and tRNA^Leu^ terminal base (*19*–*22*). The editing site of LeuRS plays a key role to maintain fidelity in protein translation by hydrolyzing tRNA^Leu^ mischarged to noncognate amino acids such as norvaline (*23*, *24*). Thus, we tested whether the benzoxaboroles **Cmpd6** and **Cmpd9** are inhibitors of *Wolbachia* LeuRS and whether these compounds compete with the main substrate of the editing site, i.e., norvaline (Fig. 1F). Norvaline is a nonproteinogenic amino acid that can be mischarged to tRNA^Leu^ in the LeuRS synthetic site and becomes toxic when incorporated into proteins, if not proofread at the LeuRS editing site (*25*). We designed constructs of the *Wolbachia* LeuRS editing domain (fig. S1), which bear the putative drug-binding pocket, for expression and purification of recombinant proteins. Two constructs gave soluble and well-behaving proteins that were used for biophysical and structural studies: a construct encoding for residues 219 to 418, herein named *Wolbachia* LeuRS; and another construct named *Wolbachia* LeuRS D1, for which part of an insertion specific to *Wolbachia* LeuRS (herein denoted as *i*1) and predicted to be highly disordered was partially deleted (354 to 377) (fig. S1). The folding and the thermal stability of both proteins were studied by differential scanning fluorimetry (DSF) in the absence and presence of compounds. Both proteins were folded, and in the presence of **Cmpd6** or **Cmpd9** with AMP, the *Wolbachian* LeuRS constructs exhibited a marked increase of the melting temperature (∆*T*_m_ = 13°C) compared to the free proteins (Fig. 1, G and H), suggesting a strong binding of the compounds to the LeuRS protein. Equivalent experiments performed in the presence of **Cmpd9** and ATP show a similar increase of the melting temperature, suggesting that ATP and AMP can form similar inhibition adducts with these compounds (fig. S2). To quantitatively assess the affinity of these compounds to *Wolbachia* LeuRS, isothermal titration calorimetry (ITC) experiments were carried out. In the absence of AMP, **Cmpd6** and **Cmpd9** did not bind to *Wolbachia* LeuRS; however, in the presence of AMP, the compounds were potent LeuRS inhibitors, with dissociation constants of 44 and 6 nM, respectively (Fig. 1, I and J). This contrasted with the one order of magnitude weaker affinity (45 μM) of the norvaline adenylate analog (Nva2AA, Fig. 1K), which represents the physiological substrate of the LeuRS editing site. **Cmpd9** has stronger affinity than **Cmpd6**, which arises from a more favorable binding entropy (table S2), likely due to a more optimal conformation by the alkyl-phenyl group of **Cmpd9** to bind into the *Wolbachia* LeuRS drug pocket.

Collectively, our experiments support a LeuRS inhibition mechanism by benzoxaboroles that is dependent on adenosine-based molecules like AMP.

### Adenosine-dependent activation mechanism of the prodrugs Cmpd6 and Cmpd9

Benzoxaborole inhibitors of *Mycobacterium tuberculosis* LeuRS that recently completed phase 2 clinical studies are prodrugs that activate with adenosine to become active (*26*). To investigate whether our anti-*Wolbachian* compounds use this activation mechanism, we used NMR spectroscopy to study whether the compounds interact with ATP or AMP in the absence of the protein (fig. S3A and B). We acquired one-dimensional (1D) ^1^H NMR spectra of free ATP and AMP and of equimolar mixtures of the compounds with AMP or ATP at cellular physiological concentrations (5 mM). The spectra show the interaction of **Cmpd6** with AMP/ATP in the absence of the protein, leading to the formation of two covalent adducts (diastereomers), differentiated by the stereochemistry of the boron atom, which we denote as adducts of type “a” (*exo-*ribose) or “b” (*endo-*ribose) (fig. S3B and C). Upon adduct formation, the boron atom changes from a neutral *sp*^2^ trigonal configuration in the free compounds to a negatively charged *sp*^3^ tetrahedral configuration in the adducts (fig. S3C).

To quantitatively assess the formation of the adenosine-based inhibitor adducts, we performed ITC experiments by titrating **Cmpd9** into AMP in the absence of protein (fig. S3D). Fitting of the experimental binding isotherm to a one-site binding model gives a *K*_d_ of 1.0 mM and shows that the interaction is mainly driven by a favorable enthalpic contribution, as expected for the formation of new bonds between the oxaborole group of the compound and the ribose hydroxyls of adenosine.

Altogether, these experiments show that benzoxaborole inhibitors of *Wolbachia* LeuRS are prodrugs that, in an enzyme-independent manner, form adducts with adenosine substrates. The adducts then bind with potent nanomolar affinity into the *Wolbachia* LeuRS editing site and efficiently compete with the physiological substrate norvaline.

### Structural basis of the inhibition of *Wolbachia* LeuRS by benzoxaborole-AMP adducts

**Fig. 2.**
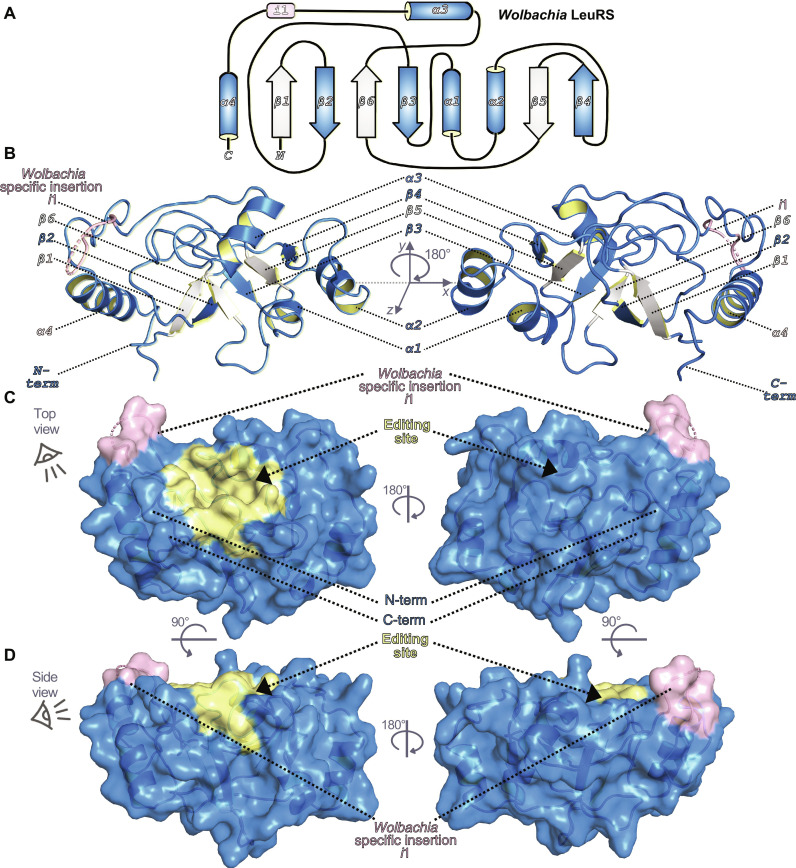
Crystal structure of apo *Wolbachia* LeuRS. (**A**) 2-D architecture of the *Wolbachia* LeuRS editing domain showing an iterative α-β secondary structure organization. For clarity, α helices are colored blue and β sheets are intermittently colored white/blue. The *Wolbachia* LeuRS specific insertion *i*1 is colored pink. (**B**) Crystal structure of *Wolbachia* LeuRS D1 editing domain shown in cartoon representation with key secondary elements colored as in (A). (**C** and **D**) Semi-transparent surface representations of the *Wolbachia* LeuRS crystal structure showing a top view rotated by 180° around the *y* axis and a side view rotated by 45° around the *x* axis, respectively. The editing site (drug-binding site) is shown in yellow and the insertion *i*1 is shown in light pink.

**Fig. 3.**
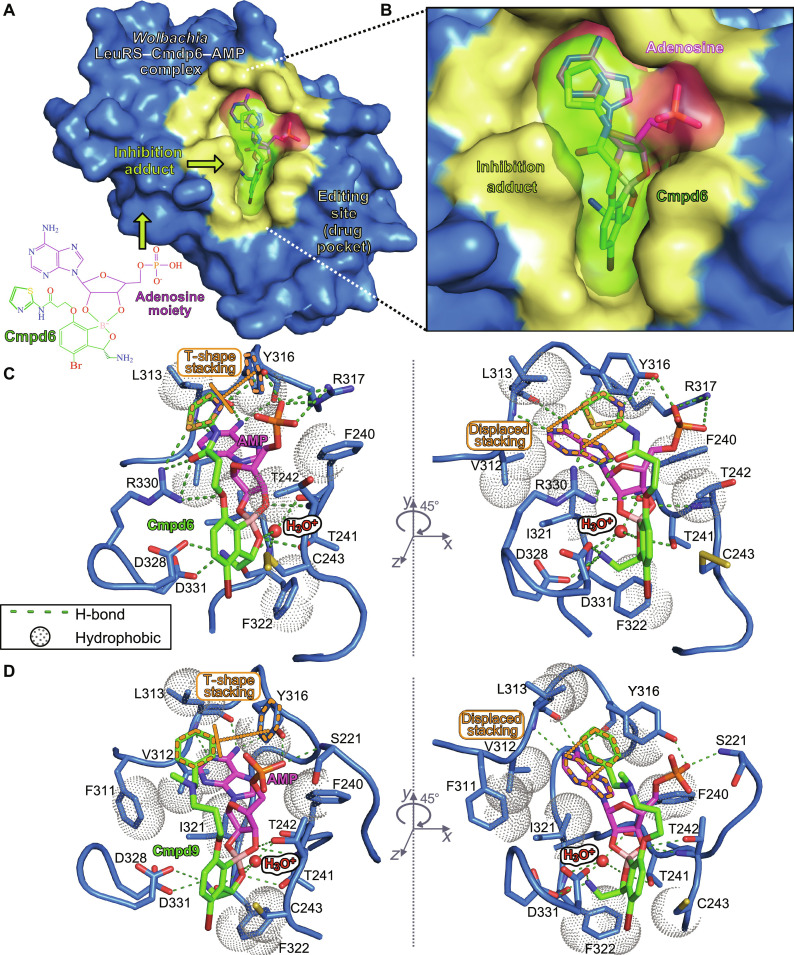
Crystal structures of the *Wolbachia* LeuRS complexes with AMP-compound adducts. (**A**) Overall structure of the *Wolbachia* LeuRS complex with the adduct AMP-**Cmpd6** bound into the editing site. Van der Waals surface representation in blue was used for the protein, with the drug-binding site in yellow. (**B**) Zoomed-in view showing the inhibition adduct in surface and stick representations, with the AMP group in pink and **Cmpd6** in green. (**C** and **D**) Main interactions established by the adenosine-drug inhibition adducts formed by **Cmpd6** and **Cmpd9**, respectively. Hydrogen bonds are shown as green dashed lines and hydrophobic interactions are shown as dotted spheres. Key protein residues are shown as blue sticks, the hydroxonium ion is shown as a red sphere, and the compound-AMP adducts are in the same color code as in (B). π-π aromatic stackings of the thiazole/phenyl rings of **Cmpd6/9** are highlighted in orange dashed lines. For clarity, panels are rotated by 45° around the *y* axis.

To unveil the structural details of the inhibition mechanism of *Wolbachia* LeuRS by these compounds, we performed crystallization trials with the protein in the presence and absence of inhibitors and AMP. By using a construct with a shortened, flexible insertion *i*1, *Wolbachia* LeuRS D1 (fig. S1), we managed to obtain high-quality crystals of the apo-protein and the complexes with the adenosine adducts formed by **Cmpd6** and **Cmpd9**, which diffracted to 3.0, 1.8, and 2.1 Å, respectively (table S3). The architecture of *Wolbachia* LeuRS consists of six antiparallel β strands that form an extended β sheet with four inserted α helices, hence adopting a β1-β2-β3-α1-α2-β4-β5-β6-α3-α4 fold (Fig. 2, A to D). Specific features of *Wolbachia* LeuRS are the very long insertion (*i*1), which connects helices α3 and α4, and two cysteines (C243 and C324) located in the drug-binding pocket, which are not found in any other bacterial LeuRS species (fig. S1, discussed later) or in human LeuRSs (cytosolic and mitochondrial). The cocrystal structures of *Wolbachia* LeuRS in complex with **Cmpd6** and **Cmpd9** show that the LeuRS inhibition mechanism relies on adenosine-based adducts of type “a,” as detected by NMR, which tightly bind into the drug pocket of LeuRS (Fig. 3, A and B, and fig. S4). Docking of diastereomers of type “b” into the *Wolbachia* LeuRS editing site shows steric clashes of the benzoxaborole within the editing site, indicating that these adducts are not LeuRS inhibitors. Docking of the active adducts of **Cmp6/9** into the editing site of human LeuRS shows substantial steric clashes (fig. S5, A to D), which explains the selectivity of this class of compounds toward bacterial LeuRS and hence their low toxicity in humans (*20*, *21*, *26*).

The potent nanomolar affinities of the two compounds are explained by a combination of multiple polar and hydrophobic interactions established by the inhibition adducts in the editing site (Fig. 3, C and D). Both compounds make equivalent interactions with *Wolbachia* LeuRS, except for minor differences in the 2-amido-thiazole and amino-methyl phenyl groups of **Cmpd6** and **Cmpd9**, respectively. Specifically, the amino-methyl phenyl of **Cmpd9** packs between residues F311, L313, and Y316, and makes several hydrophobic contacts, and at the same time, the phenyl group makes two favorable aromatic π-π interactions, one parallel-displaced with the adenosine ring and one T-shape stacking with Y316 (Fig. 3D). This conformation is further stabilized by hydrogen bonds of the adenosine-phosphate to residues Y316 and S221. In **Cmpd6**, the 2-amido-thiazole group enables multiple polar contacts to the side chains of R330 and Y316 (Fig. 3C), the latter further being stabilized by interactions of the adenosine-phosphate with Y316 and R317 and an intramolecular interaction within **Cmpd6** established by the sulfur atom with the neighboring carbonyl. Furthermore, the oxygen at position 7 hydrogen bonds to the side chain of R330 (Fig. 3C). The amido-thiazole group also packs against the adenosine ring; however, the π-π stacking is not entirely parallel compared to the phenyl of **Cmpd9**. Other key interactions, shared by the two compounds, are a hydrogen bond by the oxaborole oxygen-1 to T241; and the three hydrogen bonds established by the 3-aminomethyl to F322, D328, and D331. Moreover, a water molecule, likely activated in the form of a hydroxonium cation (H_3_O^+^), establishes four hydrogen bonds: three as a donor to the carbonyl of residues F322 and F240 and to the oxygen of the oxaborole group, and one as an acceptor to the amino group of F322 (fig. S6, A and B). This positively charged hydroxonium is likely a leaving water upon adduct formation (water 13 in the structure of the complex with **Cmpd9** and water 2 in the complex with **Cmpd6**) and fits well in a protein cavity formed by residues 239 to 241 and 321 to 322. The hydroxonium ion establishes electrostatic interactions with the negatively charged boron atom of **Cmpd6/Cmpd9** and, thus, stabilizes the tetrahedral configuration of the oxaborole moiety adopted in the inhibition adduct (fig. S6C). Altogether, the data show that the hydroxonium-mediated stabilization of the oxaborole-adenosine adduct is important for the *Wolbachia* LeuRS inhibition mechanism.

### Structural rearrangements in *Wolbachia* LeuRS upon binding of compounds

Comparison of the apo-structure to the complexes of *Wolbachia* LeuRS shows that the binding of the inhibition adducts of **Cmpd6/9** induces several conformational changes (fig. S7). Notably, the regions 267 to 270 and 280 to 285, which are not visible in the apo-structure, respectively adopt α helix and β tongue structures that stabilize the inhibition adduct in the complex (fig. S7A). In addition, the region 313 to 318, which, as judged by the high crystallographic *B* factors, is highly flexible in the apo-structure, becomes more compact in the complex and makes interactions to the adduct. Specifically, the side chain of R317 shifts 3.5 Å toward the phosphate of the **Cmpd6**–AMP adduct and stabilizes the aromatic ring of Y316, altogether enabling multiple interactions with the adduct (fig. S7B). Another key change is a 90° rotation of the R330 side chain toward the adduct that gives rise to four hydrogen bonds to the amido-thiazole group (fig. S7C), further stabilizing the inhibition adduct within the *Wolbachia* LeuRS pocket.

We also note that Cys243, which is a residue specific to *Wolbachia* LeuRS (arginine in other bacterial LeuRS pathogens) (figs. S1 and S8), forms a disulfide adduct with β-mercaptoethanol (added in our buffer) in the holo-structure with **Cmpd6** (Fig. 3C) but not in the unbound protein, suggesting that the binding of the adduct increases the reactivity of the sulfur atom of this cysteine. The sulfur is 3.5 and 3.8 Å away from carbons 5 and 6 of **Cmpd6/Cmpd9**, respectively; thus, specific 5,6-derivatized compounds have the potential to react covalently with Cys243 (fig. S8, A and B). Another residue unique to *Wolbachia* LeuRS is Cys324 (figs. S1 and S8). Cys324 does not participate in interactions; however, it is 3.7 Å away from the bromine of **Cmpd6/9**, suggesting that larger substitutions in position 4 of the core benzoxaborole could enable additional interactions (fig. S8B). In conclusion, the specificities in the *Wolbachia* LeuRS drug pocket explain the selectivity of these inhibitors, which is an important feature to advance compounds into clinical studies.

### Structural basis of the inhibition of LeuRS by oxaborole-tRNA^Leu^ complexes

**Fig. 4.**
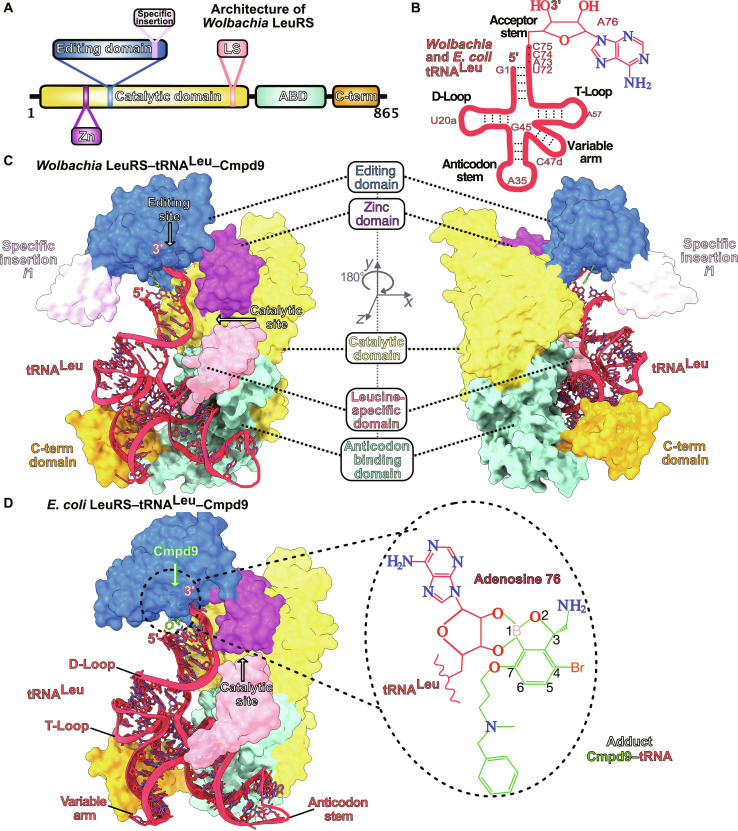
Structures of full-length LeuRS complexes with tRNA^Leu^ adenosine-compound adducts. (**A**) Domain architecture of *Wolbachia* LeuRS protein. (**B**) Cloverleaf-like secondary structures of *Wolbachia* and *E. coli* tRNAs^Leu^ with highlighted main regions. Key tRNA^Leu^ bases are numbered with the last base of the acceptor stem (Ade76) shown as sticks. (**C**) *Wolbachian* LeuRS-tRNA^Leu^ complex with **Cmpd9** built by using as templates the crystal structures of the *Wolbachia* LeuRS editing domain and of the full-length *E. coli* LeuRS-tRNA^Leu^-**Cmpd9**. The LeuRS protein is shown in surface representation, and the different domains are in the same color code as in (A). An insertion that is unique to *Wolbachia* LeuRS, named specific insertion *i1*, is shows as light-pink surface. The tRNA^Leu^ is shown in sticks/ribbon representation and **Cmpd9** is shown as green sticks. (**D**) Crystal structure of the *E. coli* LeuRS-tRNA^Leu^-**Cmpd9** complex determined at 2.1 Å resolution. Protein and tRNA^Leu^ are shown in the same color code as *Wolbachia* LeuRS complexes. The structure of **Cmpd9** is shown as green sticks. The zoomed-in view shows the adduct formed by the tRNA^Leu^ terminal adenosine with the oxaborole group of **Cmpd9**.

Next, we sought to investigate whether these *Wolbachia* LeuRS inhibitors can form tRNA^Leu^ adducts that bind into the LeuRS editing site as other 3-aminomethyl benzoxaboroles have been observed as adducts with the tRNA^Leu^ terminal base (adenosine-76). We expressed and purified full-length *Wolbachia* LeuRS protein (*Wb* FL LeuRS); however, extensive crystallization trials did not give crystals. Therefore, based on the high sequence similarity and structural homology of the LeuRS drug-binding pocket between *Wolbachia* and *Escherichia coli* as well as the tRNA^Leu^ isoacceptors (Fig. 4, A and B, and fig. S9), we produced full-length *E. coli* LeuRS (*Ec* LeuRS) and used it to model the structure of full-length *Wolbachia* LeuRS (Fig. 4C). We succeeded in obtaining high-quality crystals of the *Ec* LeuRS–tRNA^Leu^ complex with **Cmpd9**, which allowed us to determine its structure at 2.1 Å resolution (Fig. 4D). Both *Wolbachia* and *E. coli* LeuRS structures share a common domain architecture, except for an insertion of ~20 residues that is specific to *Wolbachia* (Fig. 4C and fig. S9). The cocrystal structure of the LeuRS-tRNA^Leu^ complex unambiguously shows the adduct formed by **Cmpd9** with the last base of the tRNA^Leu^ acceptor stem (Ade76) bound into the editing site (Fig. 4D). The position of the adduct formed by the terminal tRNA^Leu^ adenosine with **Cmpd9** in the complex between full-length LeuRS and tRNA^Leu^ and the interactions in the drug pocket are comparable to those observed in the *Wolbachia* LeuRS editing domain cocrystal structures with AMP-**Cmpd9** or AMP-**Cmpd6** (fig. S10). The main difference between the adducts formed by tRNA^Leu^ terminal adenosine and AMP is the orientation of the phenyl group of **Cmpd9** or, by analogy, the amido-thiazole group of **Cmpd6**. As shown above (Fig. 3, C and D), in the adduct formed with AMP, the phenyl and amido-thiazole groups stack against the adenosine base and enable multiple interactions within the *Wolbachia* LeuRS drug pocket. However, in the complex of full-length LeuRS with tRNA^Leu^ and **Cmpd9**, the phenyl group is shifted 3.5 Å away from the 3′-tRNA^Leu^ due to the intramolecular π-π stacking of the tRNA^Leu^ base Ade76 with Cyt75 (fig. S11). This increases the flexibility of the phenyl group in the adduct formed with tRNA^Leu^ compared to the AMP adduct (phenyl packed to adenosine), as reflected in the larger crystallographic *B* factors. Specifically, the phenyl moiety has significantly higher *B* factors in the tRNA^Leu^ adduct (〈*B*〉 = 59 Å^2^) compared to the rest of the adduct (〈*B*〉 = 39 Å^2^). Conversely, the *B* factors of the adduct AMP-**Cmpd9** are invariably low for all atoms, including the phenyl group. The equivalent is observed for the amido-thiazole group of **Cmpd6** in the *Wolbachia* LeuRS-AMP-**Cmpd6** crystal structure.

Thus, while the overall position of the adducts formed by tRNA^Leu^ and AMP with the compounds remains unchanged in the full-length LeuRS and editing domain complexes, the presence of tRNA^Leu^ induces a structural rearrangement of the terminal phenyl/amido-thiazole groups to make compatible the binding of the **Cmpd9/Cmpd6**-tRNA^Leu^ inhibition adducts into the LeuRS editing site.

## DISCUSSION

The strong symbiosis between parasitic worms and their *Wolbachian* microbiota, a species that is absent in humans, provides an opportunity to target this bacterium and thus to control human infections such as those caused by filarial pathogens. This is supported by the fact that some antibiotics acting on *Wolbachia* have shown promising effects in preclinical and clinical studies aiming at developing therapies to treat filarial human diseases (*5*, *27*–*29*). However, one of the main hurdles for targeting *Wolbachia* is the identification of new targets. Here, we show how the inhibition of an unprecedented target in *Wolbachia* by boron-based compounds represents an opportunity to develop therapeutics for the treatment of filarial diseases caused by parasitic worms. We identify potent anti-*Wolbachia* LeuRS inhibitor prodrugs, one of which has potent nanomolar efficacy in the standard *Wolbachian* model of infection. Our biophysical and structural analysis provides a detailed view of the inhibition mechanism, which consists of the formation of a covalent adenosine-prodrug adduct in a LeuRS-independent manner. This adduct can be formed with AMP, ATP, or tRNA^Leu^ terminal adenosine (Ade76) and binds into the *Wolbachia* LeuRS editing site with potent nanomolar affinity. Moreover, the positively charged oxaborole group of the inhibition adducts is stabilized by a negatively charged hydroxonium ion that fits well in a cavity of the *Wolbachia* LeuRS binding site.

The new high-resolution crystal structures presented reveal idiosyncratic features at the drug binding pocket, which rationalize the selectivity of these *Wolbachia* inhibitors. These features enable the development of narrow-spectrum anti-infectives targeting *Wolbachia*, a much-heralded property for the development of new antimicrobials with minimized dysbiosis in patients.

In conclusion, LeuRS is a valid drug target of *Wolbachia*, and besides the recently identified anti-*Wolbachian* compounds such as flubentylosin, azaquinazoline derivatives, and boron-pleuromutilins (*27*, *29*–*31*), the benzoxaborole inhibitors revealed here provide a new approach for the treatment of filarial diseases. More generally, this study demonstrates the benefits of selective disruption of bacterial microbiota in strong symbiosis with host pathogens causing disease in humans, a therapeutic approach with vast potential for other infectious diseases. As research continues to unravel the intricacies of the microbiota, we can anticipate transformative breakthroughs that will revolutionize the way we control infectious diseases.

## MATERIALS AND METHODS

### Efficacy of compounds against *Wolbachia* in an insect cell infection model

The efficacy of compounds, determined as EC_50_ against *Wolbachia* in infected host cells, was determined through the anti-*Wolbachia* consortium’s (A·WOL) routine screening assay as described previously (*18*, *27*, *29*). Briefly, the cell line C6/36 derived from the mosquito vector of disease *Aedes albopictus*, stably infected with *W. pipientis* (*w*AlbB) [C6/36 (*w*AlbB)], was incubated with the compounds of interest in a dose range from 5 μM to 10 nM. Compounds were incubated for 7 days with 2000 cells per well on a 384-well plate (CellCarrier-384 Ultra, PerkinElmer) in Leibovitz media (Life Technologies) supplemented with 20% fetal bovine serum (FBS, Thermo Fisher Scientific), 2% tryptose phosphate broth (Sigma-Aldrich), and 1% nonessential amino acids (Sigma-Aldrich). The endpoint readout utilized DNA staining of both the host cell nuclei and intracellular *Wolbachia* (SYTO 11) combined with a high content imaging system (Operetta, PerkinElmer) and analyzed using the associated Harmony software through a cytoplasm texture analysis. Stock solutions at 10 mM of chemical compounds were prepared in dimethyl sulfoxide (DMSO). Chemical compounds were provided by chemical manufactures (ChemPartner and Anacor Pharmaceuticals), and quality control was verified by NMR spectroscopy (^1^H-NMR) and liquid chromatography coupled to mass spectrometry. Compound purity was higher than 95%. Compound identity and chemical structures are summarized in table S1.

### Expression and purification of full-length *Wolbachia* LeuRS for in vitro activity assays

The full-length *Wolbachia* LeuRS construct corresponds to residues 1 to 865 of *Wolbachia* sp. *Brugia malayi* (Uniprot Q5GS31). It was sub-cloned into a pET-16b vector (Genscript) at the restriction sites Nde I and Bam HI, which contains an N-terminal hexahistidine tag followed by a tobacco etch virus (TEV) cleavage site. The protein was overexpressed in *E. coli BL21-CodonPlus* (*DE3*)*-RIL* grown in terrific broth medium at 37°C until the optical density at 600 nm reached 0.9. Bacerial cell cultures were cooled down to 23°C on a water/ice bath and protein expression was induced with the addition of 1 mM IPTG and then incubated overnight at 23°C. The cells were collected by centrifugation and frozen at −80°C. Cell lysis was carried out by sonication using as purification buffer 20 mM Tris-HCl, pH 8.5, 100 mM NaCl, 5 mM MgCl_2_, and 20 mM β-mercaptoethanol supplemented with protease inhibitor tablets (Roche). The protein was purified by nickel (Ni-NTA) affinity chromatography (Qiagen). Three washing steps of 50 ml each with buffer were used for Ni affinity chromatography with the same buffer as the purification buffer with the addition of (i) 15 mM imidazole, (ii) 1 M NaCl, and (iii) 15 mM imidazole. The elution buffer was the same as the purification buffer with the addition of 500 mM imidazole. The protein was further purified by size-exclusion chromatography using a HiLoad 200 Superdex column (AKTA) equilibrated in the buffer 20 mM Tris-HCl, pH 8.5, 100 mM NaCl, 5 mM MgCl_2_, and 5 mM dithiothreitol supplemented with protease inhibitor tablets (Roche). The full-length *Wolbachia* LeuRS protein was concentrated to 10 mg/ml with a 50-kDa cutoff membrane (Millipore) and flash-frozen in liquid nitrogen before storage at −80°C.

### Anti-*Wolbachia* LeuRS in vitro activity of compounds

The IC_50_ values were calculated using a similar approach as described earlier (*19*, *20*). Briefly, *Wolbachia* full-length LeuRS, total *E. coli* tRNA crude extract (Roche), and compounds were preincubated for 20 min in 50 mM Hepes-KOH (pH 8.0), 30 mM MgCl_2_, 30 mM KCl, 0.02% (wt/vol) bovine serum albumin, and 1 mM dithiothreitol with 20 μM [^14^C]leucine (306 mCi/mmol; PerkinElmer) at 30°C. Reactions were started by the addition of 4 mM ATP and, at specific times, tRNA was precipitated by the addition of 10% (w/v) trichloroacetic acid (TCA), recovered by filtration (Millipore Multiscreen; MSHAN4B50), washed with 5% TCA (w/v), and counted by a liquid scintillation counter (Wallac). All reactions were performed in triplicate, and the mean values were used to determine an IC_50_ using Prism 4 (GraphPad). The reactivation rates (*T*_½_) were determined by measuring the time required for *Wolbachia* LeuRS to recover 50% of its activity. Full-length *Wolbachia* LeuRS (40 nM) and tRNA and compounds were preincubated for 1 hour at 4°C to inhibit 90% of the enzyme’s activity (approximately 10 times the value of the [IC_50_]). LeuRS inhibitor complexes were then diluted 200-fold, and enzyme activity was determined at various time intervals, as described before. Compound reactivation rates were determined by fitting the LeuRS activity reactivation curves to a one-phase exponential decay model (GraphPad Prism).

### Expression and purification of *Wolbachia* and *E. coli* LeuRS samples and in vitro transcription of *E. coli* tRNA^Leu^

The *Wolbachia* LeuRS constructs used for crystallization correspond to residues G219-I418 of *Wolbachia* sp. *Brugia malayi* (Uniprot Q5GS31), and the *Wolbachia* LeuRS D1 is the same, but it has a deletion of region I354-N377. It was sub-cloned into a pET-M11b vector (EMBL), which contains an N-terminal hexahistidine tag followed by a TEV cleavage site. The protein was overexpressed in *E. coli BL21-CodonPlus (DE3)-RIL* grown in lysogeny broth (LB) medium at 37°C until the optical density at 600 nm reached 0.6. Protein expression was induced with the addition of 0.4 mM IPTG and then incubated overnight at 18°C. The cells were collected by centrifugation and frozen at −80°C. Cell lysis was carried out by sonication using as purification buffer 20 mM Tris-HCl, pH 7.5, 100 mM NaCl, and 5 mM β-mercaptoethanol supplemented with protease inhibitor tablets (Roche). The protein was purified by nickel (Ni-NTA) affinity chromatography (Quieten). The washing buffer used for Ni-NTA chromatography was the same as the purification buffer with the addition of 10 mM imidazole and 1 M NaCl. The elution buffer was the same as the purification buffer with the addition of 400 mM imidazole. The His-tag was cleaved using 1 mg of TEV protein per 100 mg of His-tag protein and dialyzed for 1 hour at 4°C against the purification buffer. The protein was further purified by size-exclusion chromatography using a HiLoad 16/600 Superdex column (Cytiva) equilibrated in the purification buffer. *Wolbachia* LeuRS D1 protein was concentrated to 15 mg/ml and flash-frozen in liquid nitrogen before storage at −80°C.

The full-length *E. coli* LeuRS was overexpressed using *E. coli BL21-CodonPlus (DE3)-RIL* competent cells and purified by Ni-NTA chromatography as previously described (*19*). The *E. coli* tRNA^Leu^ was produced by in vitro transcription and purified by polyacrylamide gel electrophoresis followed by diethylaminoethyl (DEAE) Sepharose chromatography according to a previously described protocol (*19*).

### Crystallization of protein(s), tRNA, and small-molecule inhibitors

All crystallizations were performed at 20°C by the hanging-drop vapor diffusion method, using 24-microwell plates, by mixing equal volumes of protein solution and reservoir solution. Samples for crystallization were made of *Wolbachia* LeuRS D1 at 15 mg/ml, AMP at 10 mM, and 1 mM **Cmpd6/9**, or of *Ec* LeuRS full-length at 33 μM, tRNA^Leu^ at 40 μM, and 1 mM **Cmpd6/9**. They crystallized respectively in 0.1 M Tris (pH 8.5), 0.2 M lithium sulfate, and 31 to 33% (w/v) polyethylene glycol, molecular weight 4000 (PEG 4000); and in 0.1 M sodium acetate (pH 5.6), 0.2 M sodium chloride, and 16 to 20% (w/v) PEG 6000. The crystals were transferred for a few seconds in the mother liquor containing 22% (v/v) ethylene glycol as cryoprotectant and then frozen in liquid nitrogen. Crystals were diffracted at the European Synchrotron Radiation Facility (ESRF) beamlines ID23-1, ID23-2, ID29, and ID30A-1 (Grenoble, France). Data collection parameters and refinement statistics are summarized in table S3.

### Structure determination

Data reduction was carried out by XDS and autoPROC (Global Phasing). Phaser (CCP4 v. 8.0.009) was used to perform molecular replacement using PDB code 3ZJV (*20*) as a model for the full-length *E. coli* structures and *Streptococcus pneumoniae* LeuRS editing domain [PDB: 4K47 (*22*)] as a model for *Wolbachia* LeuRS editing domain structures. Manual building of several protein and tRNA regions in Coot (v. 0.9.8.5) (*32*) was used to complete the final model, and refinement was carried out with Refmac5 (CCP4 v. 8.0.009) (*33*) and Buster (Global Phasing). Validation of the models was performed with MolProbity (*34*). Figures were prepared with PyMOL (v2.5.4, Schrödinger), ChimeraX (*35*), and protein-drug interaction schemes by LigPlot+ (*36*).

### Isothermal titration calorimetry

ITC measurements were performed on a MicroCal iTC200 (GE Healthcare, PA) at 25°C. *Wolbachia* LeuRS samples were previously dialyzed into the ITC buffer 50 mM Hepes-KOH, pH 7.5, 30 mM KCl, 30 mM MgCl_2_, and 5 mM β-mercaptoethanol and adjusted to a final concentration of 70 μM. Dialysis buffer was filtered at 0.45 μm and used to dissolve AMP at 10 mM and compounds **Cmpd6** and **Cmpd9** (ChemPartner Europe) at 1 mM final concentration. A typical ITC experiment consisted of 26 injections of 1.5 μl every 180 s at a stirring speed of 750 rpm. The raw data were processed using MicroCal PEAQ-ITC (v. 1.0.0.1259, Malvern) and Microcal Origin for ITC (v. 7.0). Thermodynamic binding parameters are summarized in table S2.

### Thermal shift assays

The thermal shift assays (TSAs) were performed using a qPCR CFX96 (Bio-Rad) with the FRET scan mode. Samples were prepared in a TSA buffer containing 50 mM Hepes (pH 7.5) and 150 mM NaCl. Each well contained 2 μl of samples as prepared for the crystallization trials, 18 μl of TSA buffer, and 20 μl of 10× SYPRO orange (S6650, Thermo Fisher Scientific). The melting curve was obtained by heating samples from 25° to 95°C at a constant rate of 1°C/min.

### NMR spectroscopy

NMR samples of AMP, ATP, AMP + **Cmpd6**, and ATP + **Cmpd6** were prepared in 50 mM phosphate buffer, 30 mM KCl, and 30 mM MgCl_2_ in 99% D_2_O at pH 7.0 (meter reading at 20°C). In all cases, the concentrations of the samples were 5 mM and mixtures of AMP/ATP with benzoxaboroles were prepared in a 1:1 molar ratio. 1D ^1^H NMR spectra and 2D ^1^H-^1^H Total Correlation Spectroscopy (TOCSY) spectra were acquired for each sample at 25°C on a Bruker spectrometer operating at a ^1^H frequency of 850 MHz. The TOCSY spectra were recorded with a mixing time of 80 ms and used for assignments of the 1D spectra.
