# Supplementary material for: Targeting a microbiota Wolbachian aminoacyl-tRNA synthetase to block its pathogenic host
Source: Sci Adv. 2024 Jul 10;10(28):eado1453. doi: 10.1126/sciadv.ado1453 (PMC11235159; doi:10.1126/sciadv.ado1453)
Supplement: Supplementary file 1 — Figs. S1 to S11 Tables S1 to S3 [file sciadv.ado1453_sm.pdf]

Science Advances
AAAS

Supplementary Materials for

Targeting a microbiota *Wolbachian* aminoacyl-tRNA synthetase to block its pathogenic host

Guillaume Hoffmann *et al.*

Corresponding author: Andrés Palencia, andres.palencia@inserm.fr

Sci. Adv. **10**, eado1453 (2024)
DOI: 10.1126/sciadv.ado1453

This PDF file includes:

Figs. S1 to S11
Tables S1 to S3

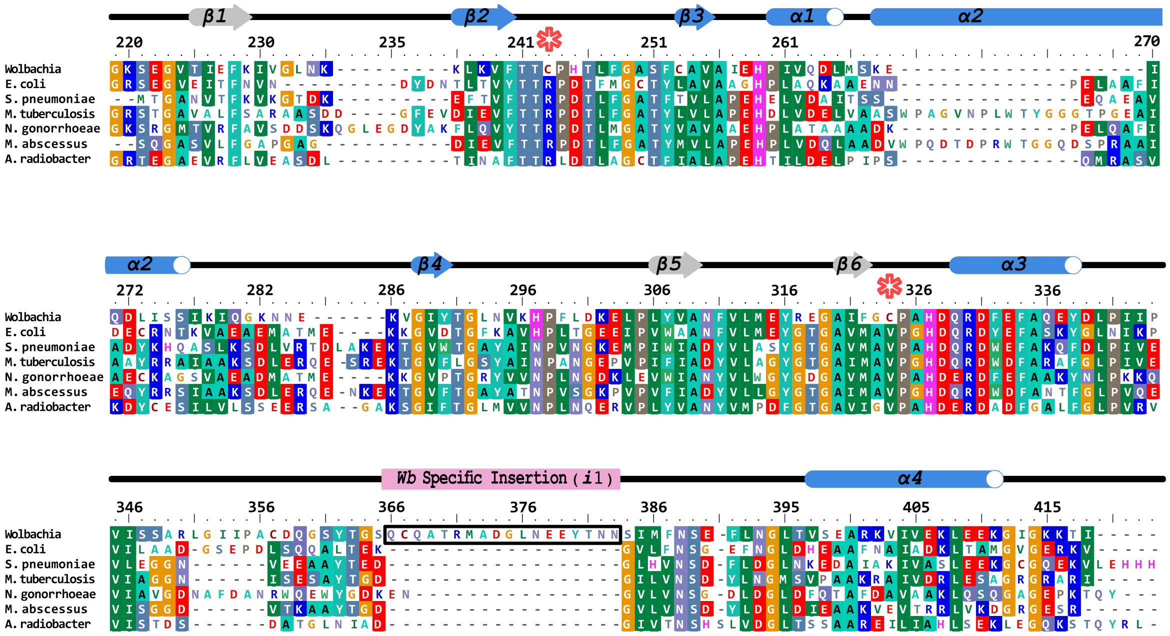

Fig. S1. Bacterial LeuRS sequence alignments and predicted structural architecture of *Wolbachia* LeuRS showing specific structural motifs. The predicted sequence of the editing domain of *Wolbachia* LeuRS was aligned to representative LeuRS sequences for which experimentally determined structures are available at the Protein Data Bank (PDB). Secondary structural elements are shown on the top of the *Wolbachia* LeuRS sequence. Idiosyncratic elements of *Wolbachia* LeuRS are the long specific insertions (herein named as *i*1, colored in pink) and cysteines 243 and 324 (depicted by red asterisks). The two *Wolbachia* LeuRS editing domain constructs used in this study were *Wolbachia* LeuRS (219–418), and *Wolbachia* LeuRS D1 (219–418 with the deletion 354–377).

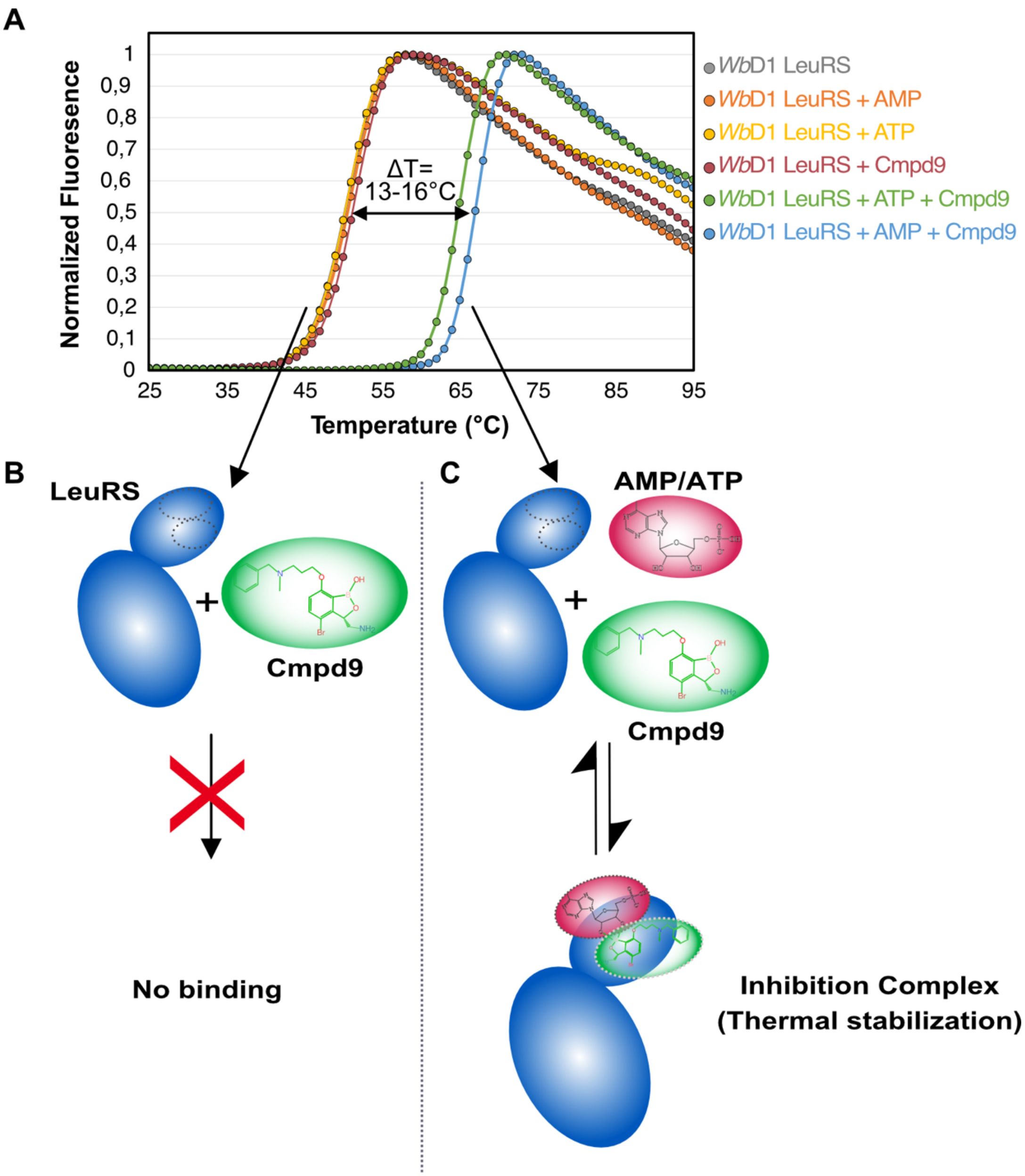

Fig. S2. Stabilization of *Wolbachia* LeuRS in the presence of Cmpd9 with ATP or AMP. (A) Thermal-shift assays showing the thermal stability of *Wolbachia* LeuRS protein alone; in the presence of the inhibitor **Cmpd9**; in the presence of ATP or AMP; and in the presence of **Cmpd9** + ATP or AMP. The plotted curves represent the average of experiments were performed in triplicates. **(B)** Schematic diagram showing that the absence of ATP or AMP does not allow the formation of the inhibition complex. **(C)** In the presence of adenosine-based biomolecules like

ATP or AMP, an inhibition adduct is formed by **Cmpd9** which binds into the *Wolbachia* LeuRS editing site and leads to thermal stabilization of the protein.

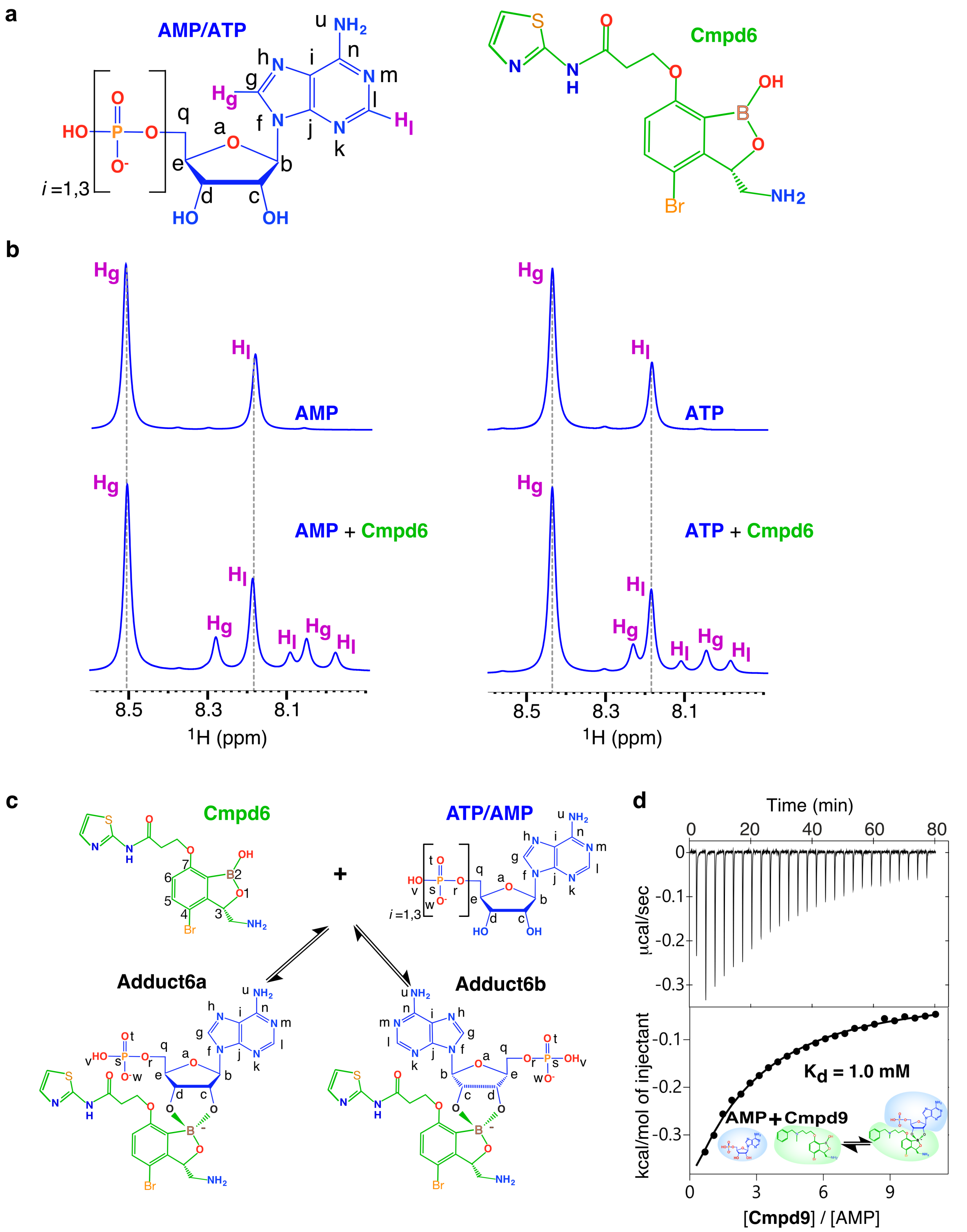

Fig. S3. Cmpd6 and Cmpd9 are prodrug inhibitors of LeuRS that activate by forming adducts with ATP/AMP in an enzyme-independent manner. (A) Chemical structures of ATP/AMP and **Cmpd6** with the atom nomenclature used in NMR experiments. **(B)** ^{1}H NMR

spectra of AMP/ATP alone or in equimolar (5 mM) mixtures with **Cmpd6**. A zoomed-in view on the aromatic NMR signals of AMP/ATP is shown. Upon addition of the compounds, the NMR signals of the aromatic groups of AMP/ATP split into an additional two peaks corresponding to the two adducts formed. **(C)** Spontaneous cyclization of **Cmpd6** with ATP/AMP leading to the formation of two diastereomer adducts based on the pseudo symmetry of the ribose. For convenience, adducts with an *exo*-ribose in panel c) are denote as type “a”, and those with *endo*-ribose as adducts of type “b”. **(D)** Isothermal Titration Calorimetry experiment with **Cmpd9** injected into AMP leading to the formation of adducts by **Cmpd9**. An equivalent binding isotherm curve was observed with **Cmpd6** and AMP. The experiment was performed in triplicate.

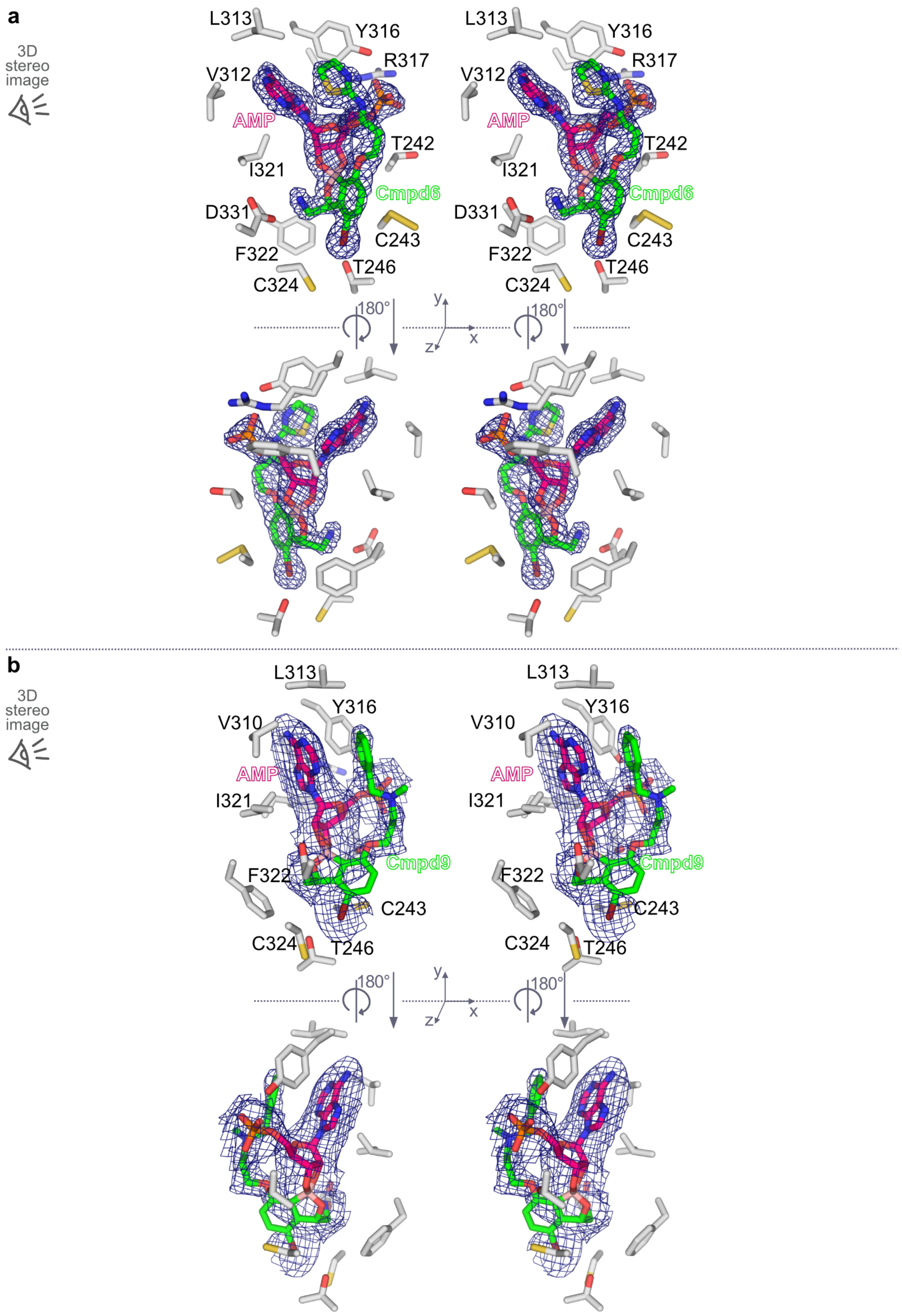

Fig. S4. Stereo view of the *Wolbachia* LeuRS drug pocket with unbiased electron density of inhibition adducts. (A) The electron density omit maps (Fo – Fc) calculated for AMP adducts with **Cmpd6** and **(B) Cmpd9** are contoured at five and two sigma, respectively and shown as blue

grids. **Cmpd6** and **Cmpd9** are shown as green sticks, adenosine-adduct moiety as pink sticks, and protein residues as white sticks. For clarity, bottom panels are rotated 180° with respect to top panels. Stereo view can be facilitated by the use of stereoscopic glasses.

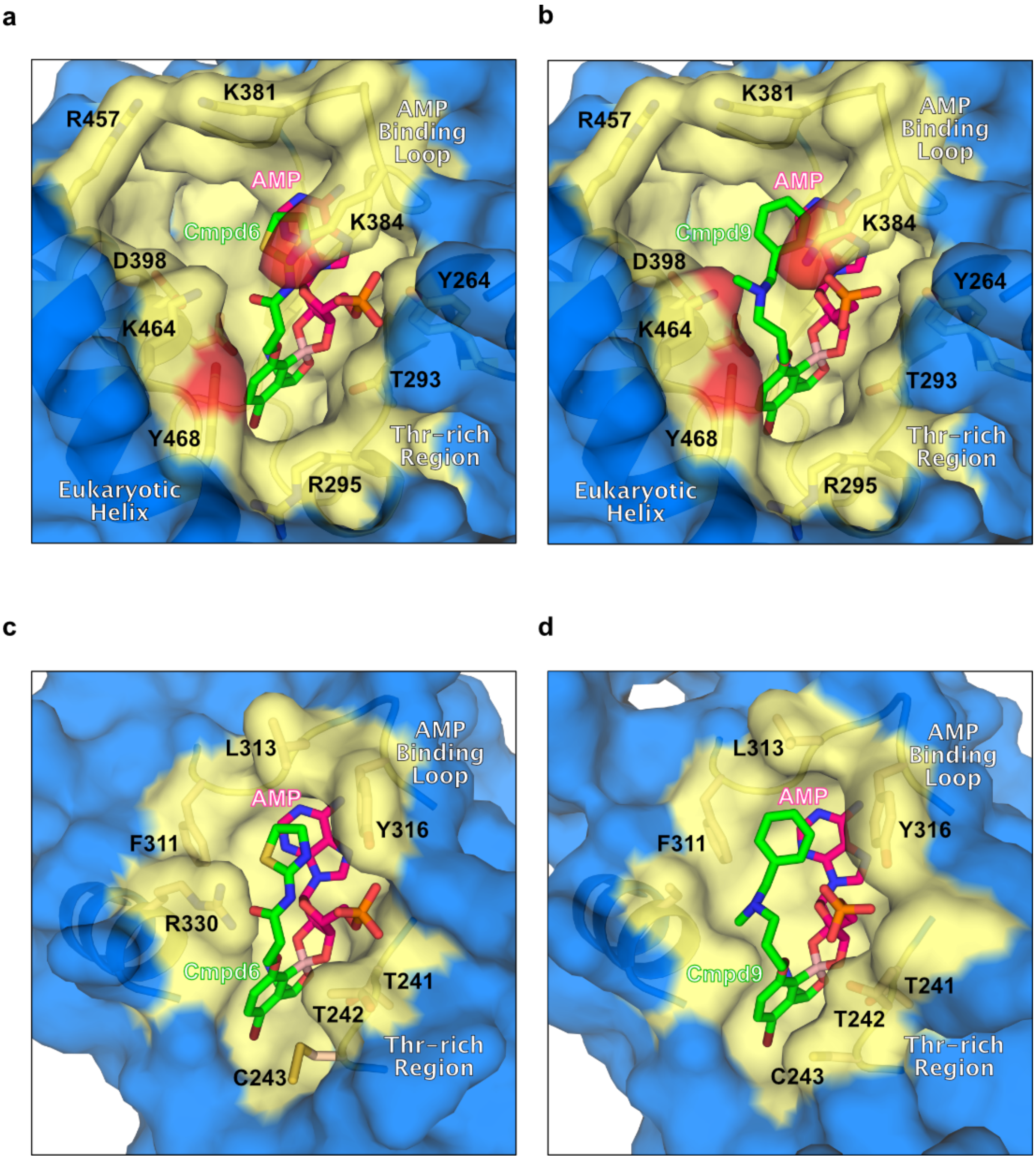

Fig. S5. Benzoxaborole inhibition adducts are not competent for binding to the human LeuRS. (A), (B) Surface representation of the human cytosolic LeuRS editing domain (PDB ID: 2WFD) docked with the active AMP–adducts formed by **Cmpd6** and **Cmpd9** respectively. Protein is shown as a blue cartoon over semi-transparent surface representations with the drug-binding pocket in yellow. Key protein residues are shown as yellow sticks. **Cmpd6/9** adducts are shown in sticks with the adenosine moiety in pink and the compound in green. Steric clashes of compound–AMP adducts to human LeuRS editing site are shown in red, and consists mainly of

clashes with the eukaryotic helix and the AMP-binding loop. The different geometry of the human LeuRS pocket has poor surface complementarity with the adducts, which leads to the loss of Van der Waals and hydrophobic interactions in the above-mentioned regions as well as the Thr-rich region. For comparison, panels **(C)** and **(D)** show the same orientation of the crystal structures of *Wolbachia* LeuRS in complex with AMP adducts formed by **Cmpd6** and **Cmpd9**, respectively. Residue R330 is only partially visible in the complex of *Wolbachia* LeuRS with **Cmpd9**.

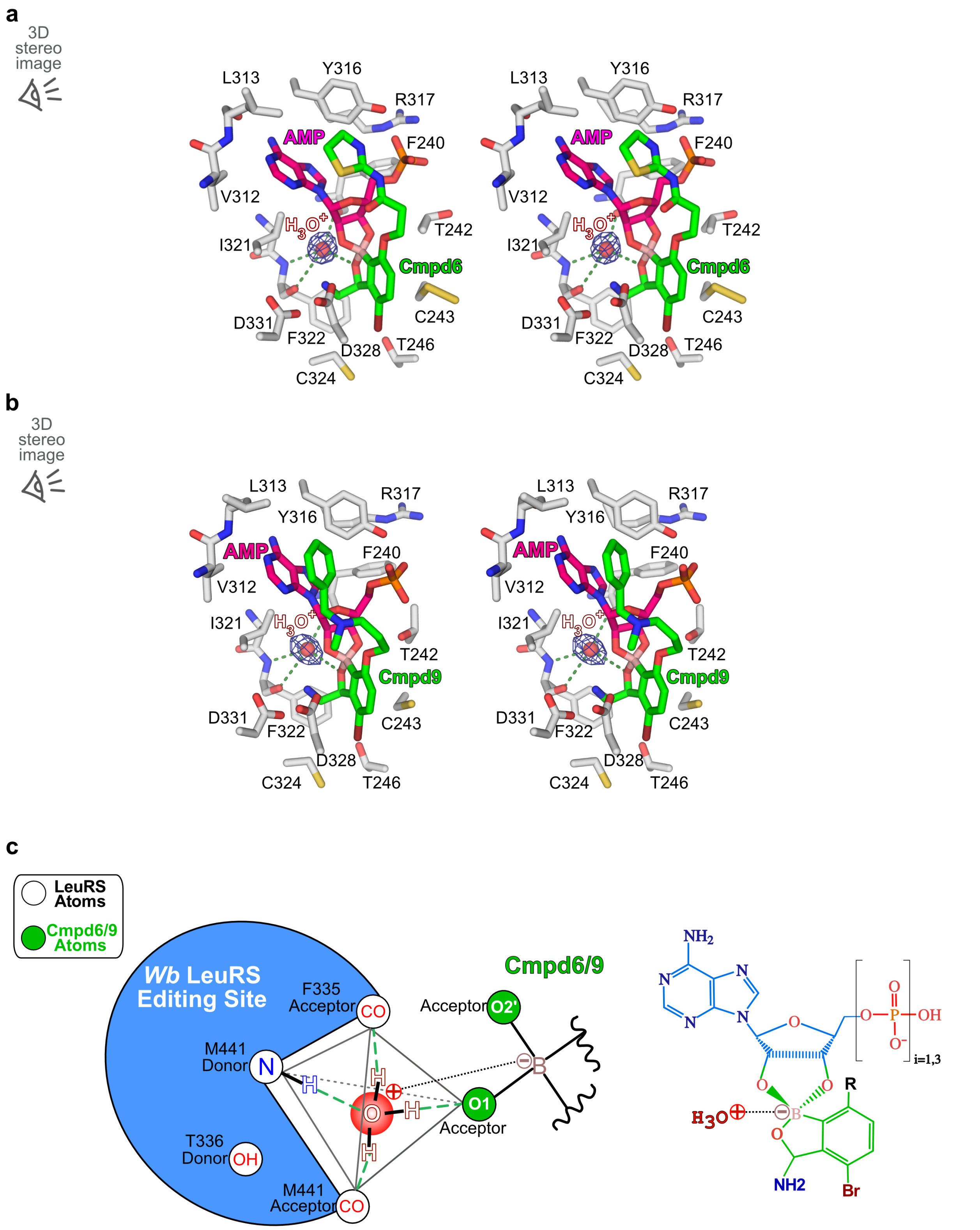

Fig. S6. A hydroxonium ion stabilizes the inhibition adduct in the *Wolbachia* LeuRS drug site. (A) Stereo view of the electron density omit maps (Fo – Fc) calculated for hydroxonium ions in the complex with AMP–**Cmpd6** and **(B)** AMP–**Cmpd9**. Electron density maps were shown as

blue grids and contoured at four- and three-point five sigma for AMP–**Cmpd6** and AMP–**Cmpd9** complexes, respectively. **Cmpd6** and **Cmpd9** are shown as green sticks, adenosine-adduct moiety as pink sticks, and protein residues as white sticks. **(C)** Schematic representation of the hydrogen bonds established by the hydroxonium ion in the *Wolbachia* LeuRS drug-binding pocket (shown as blue surface). The four observed hydrogen bonds and their pyramidal configuration are indicative of hydroxonium ions. Protein atoms are labelled and represented as white spheres, whereas compound atoms are shown as green spheres. The charged hydroxonium ion is shown as a red sphere. Besides the main donor and acceptor atoms establishing hydrogen bonds to the adduct, other surrounding atoms with hydrogen bonding potential are also shown. For clarity, the chemical structure of the whole inhibition adduct AMP–**Cmpd6/9** is shown in the right panel.

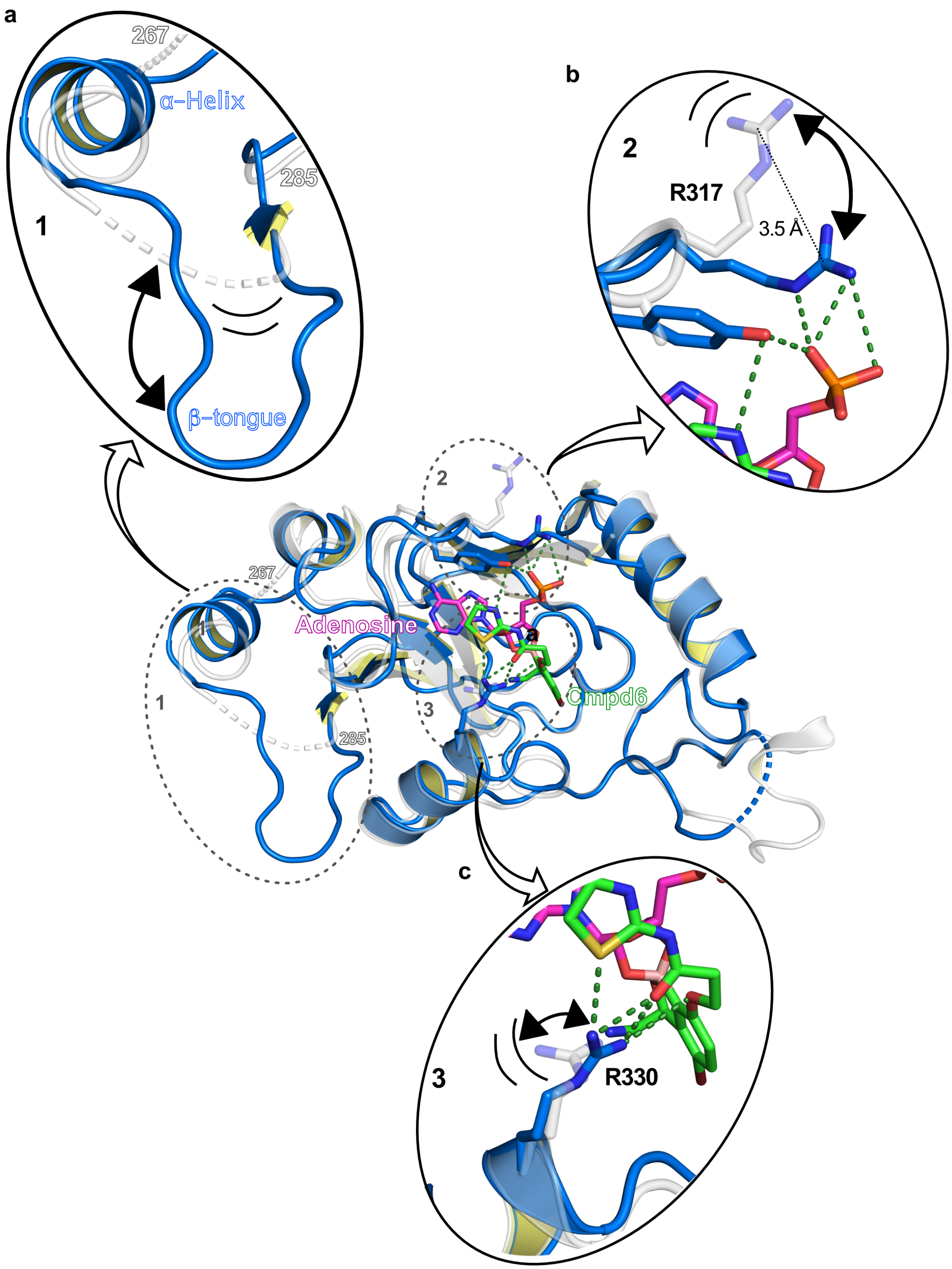

Fig. S7. Conformational changes induced by the binding of the AMP–Cmpd6/9 inhibition adducts to the *Wolbachia* LeuRS editing site. (A) The region 267-285 that is highly dynamic and not visible in the apo-structure folds into an alpha-helix and a beta-tongue upon binding of the **Cmpd6**–AMP adduct. The *Wolbachia* LeuRS apo-structure is shown as white cartoon and stick

representation, and hydrogen bonds as dashed green lines. **(B)** The binding of the inhibition adduct induces the rearrangement of Arg317 enabling three hydrogen bonds to the adduct, as well as Tyr316 (not visible in the apo-structure) that reinforces the interaction to the adduct with two additional hydrogen bonds. **(C)** The re-orientation of Arg330 upon the binding of the inhibition adduct allows the formation of several hydrogen bonds to atoms of **Cmpd6**, further anchoring the inhibition adduct into the editing site.

a

b

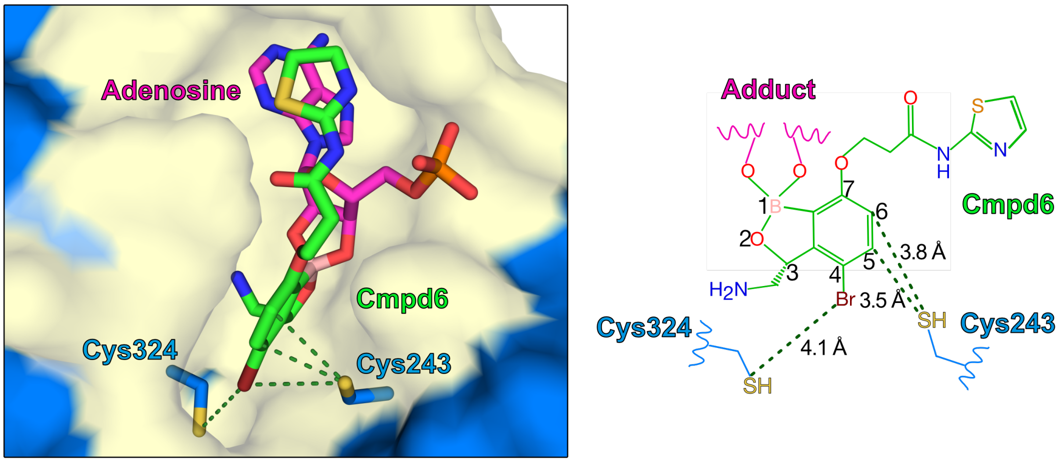

Fig. S8. Cysteines 243 and 324 are *Wolbachia*-specific residues. (A) Zoomed-in view of the adduct **Cmpd6**–AMP bound into the *Wolbachia* LeuRS editing site. The protein drug-binding pocket is highlighted in yellow and the cysteines 243 and 324 are shown as blue sticks with sulphur atoms in yellow. The AMP moiety is colored in pink and **Cmpd6** in green. **(B)** Schematic representation of **Cmpd6** and *Wolbachia* LeuRS Cys243 and Cys324 with distances of the sulphur cysteine atoms to key positions of the inhibitors. The compound positions 4, 5 and 6 have the potential of being functionalized to enable covalent bond formation to sulphur cysteines, which provide alternative possibilities, besides the current successful one, to get selectivity of inhibitors *Wolbachia* LeuRS.

a

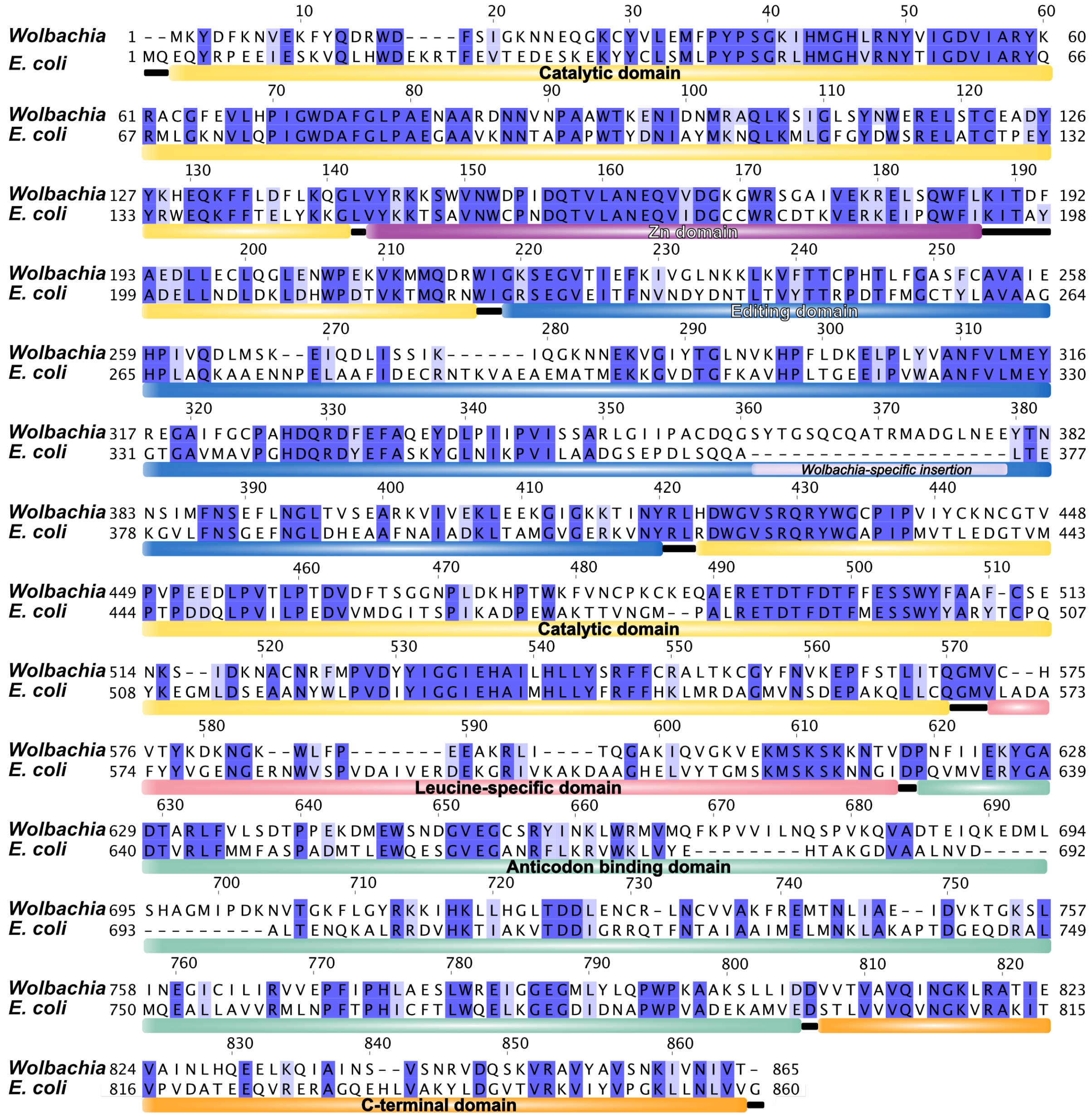

Fig. S9. Sequence alignment of full-length *Wolbachia* and *E. coli* LeuRS protein sequences. The shown sequence alignment, the experimentally determined structures of the editing domain of *Wolbachia* LeuRS and the one of *E. coli* LeuRS–tRNA^{Leu}–**Cmpd9** were used to build the model of full-length *Wolbachia* LeuRS–tRNA^{Leu}–**Cmpd6/9** complexes. The *Wolbachia* LeuRS domain architecture was calculated based on the *E. coli* LeuRS structures, with all the different domains labelled and shown with the same colour code as in **Fig. 4**. Strictly conserved residues are highlighted with dark blue in the sequence alignment, and semiconserved residues as light blue.

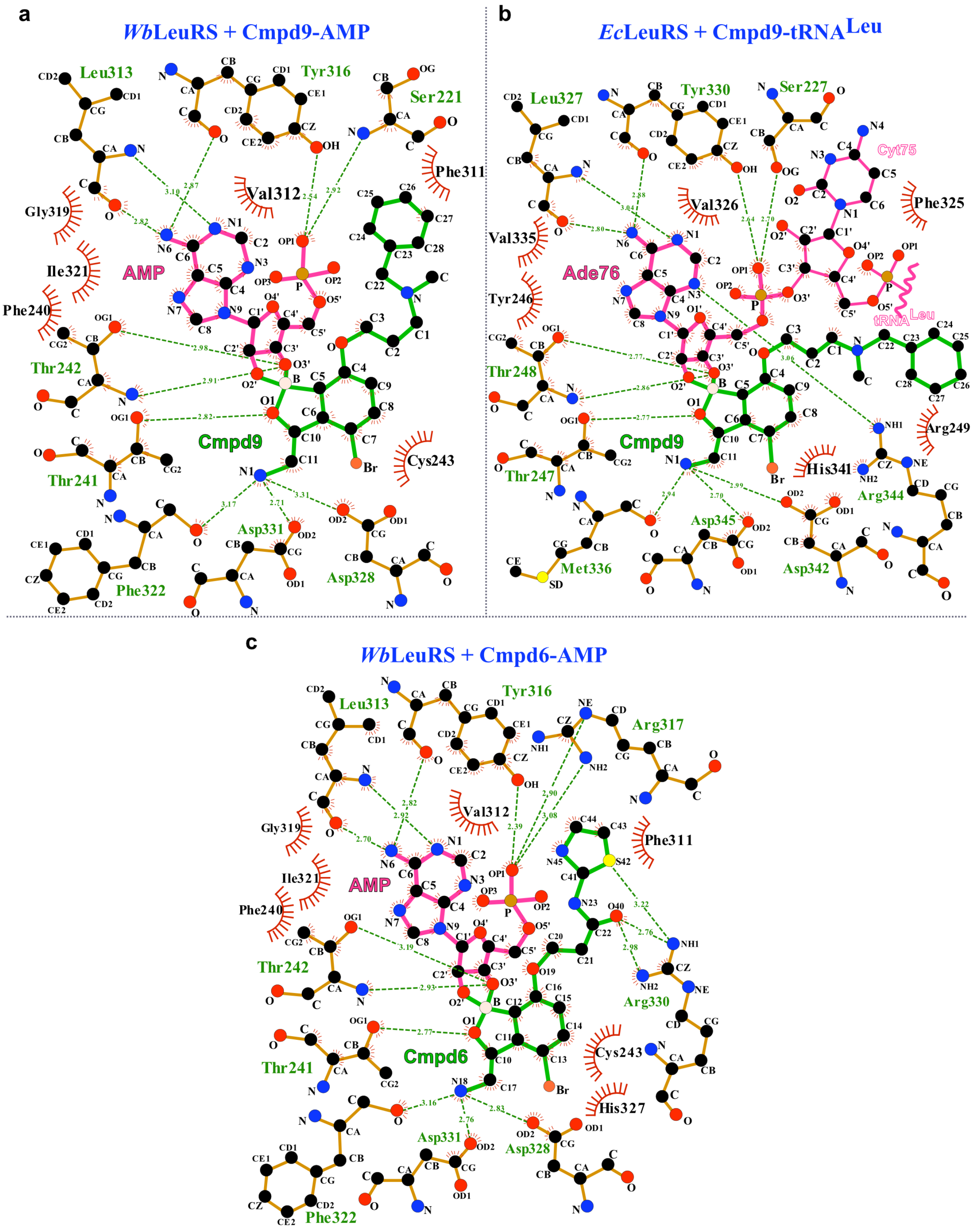

Fig. S10. Detailed analysis of the interactions established by compound-adducts in *Wolbachia* and *E. coli* LeuRS complexes. (A) *Wolbachia* LeuRS complex with the inhibition adduct

Cmpd9–AMP bound in the LeuRS editing site. Hydrogen bonds are shown as green-dashed lines and hydrophobic interactions as dashed red semi-circles. The adenosine structure is shown as pink sticks and the compounds as green sticks. Protein residues are shown as orange sticks. The same colour code is used in all panels. **(B)** *E. coli* LeuRS–tRNA^{Leu} complex with the inhibition adduct formed by **Cmpd9** with tRNA^{Leu} base Ade76 bound in the LeuRS editing site. For reference, tRNA^{Leu} base Cyt75 was also shown. **(C)** *Wolbachia* LeuRS complex with the inhibition adduct **Cmpd6**–AMP bound in the LeuRS editing site.

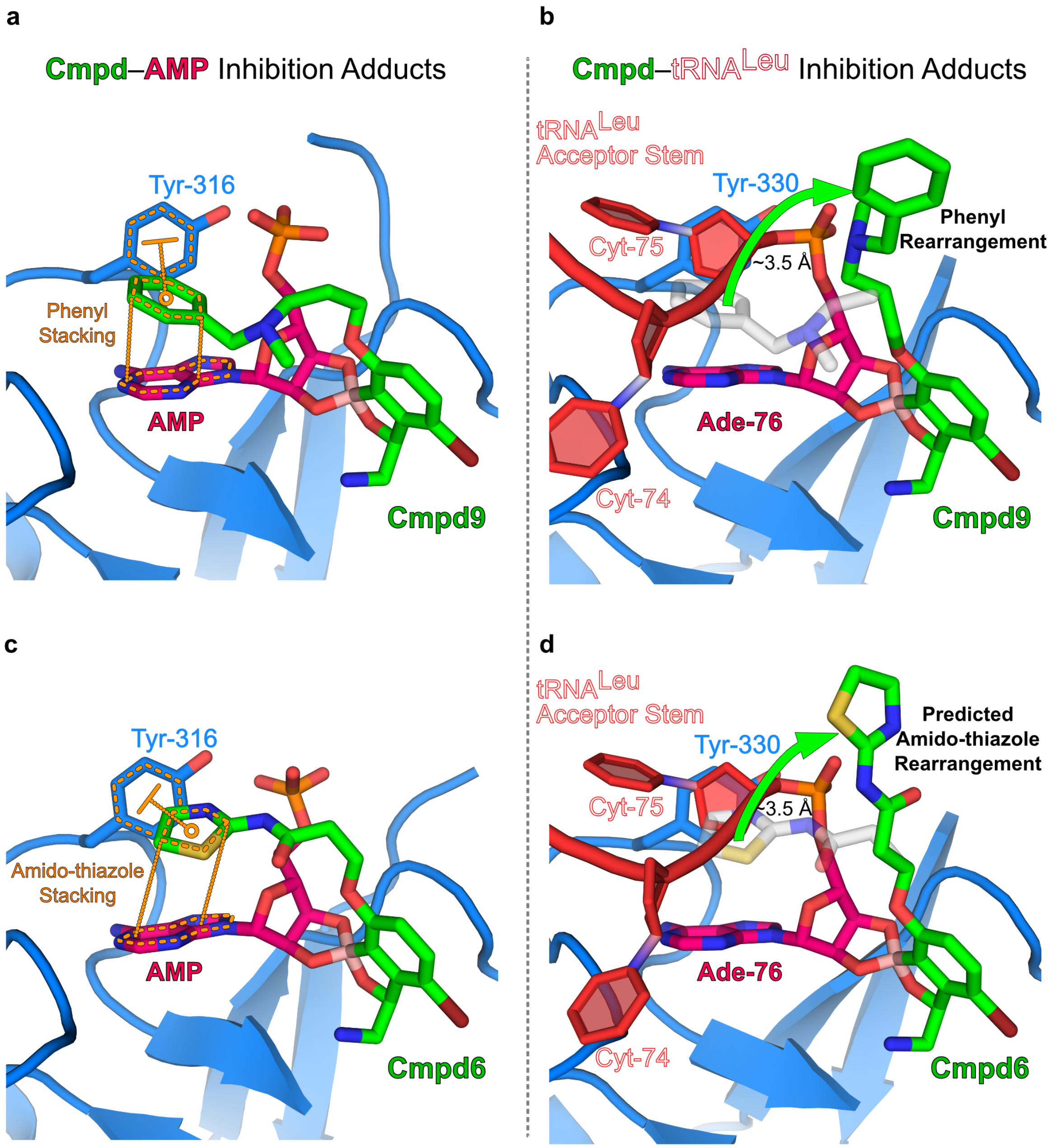

Fig. S11. Structural differences of compound adducts formed with AMP or tRNA^{Leu}. (A) *Wolbachia* LeuRS complex with the inhibition adduct **Cmpd9**–AMP bound in the LeuRS editing site. The phenyl group of **Cmpd9** forms two types of aromatic π-π interactions: one T-shape stacking with Tyr316 and a displaced-parallel stacking with adenosine. Stackings are highlighted in orange, protein as blue cartoon/sticks, and AMP-Cmpd adduct with green sticks for **Cmpd9** and pink sticks for AMP. The same colour code is used in all panels. **(B)** *E. coli* LeuRS–tRNA^{Leu} complex with the inhibition adduct formed by **Cmpd9** with tRNA^{Leu} base Ade76 bound in the

LeuRS editing site. The presence of Cyt75 induces a shift of the phenyl ring, which can no longer stack with adenosine 76 and Tyr330. The $tRNA^{Leu}$ Cyt74 and Cyt75 are shown as filled-red bases. **(C)** *Wolbachia* LeuRS complex with the inhibition adduct **Cmpd6**–AMP bound in the LeuRS editing site. The amido-thiazole ring of **Cmpd6** forms two types of aromatic π-π interactions: one T-shape stacking with Tyr316 and a displaced-parallel stacking with the adenosine. **(D)** *E. coli* LeuRS–$tRNA^{Leu}$ complex with the inhibition adduct formed by **Cmpd6** with $tRNA^{Leu}$ base Ade76 docked into the LeuRS editing site. The presence of Cyt75 would induce a shift of the amido-thiazole group, which can no longer stack with adenosine 76 and Tyr330. The position of the $tRNA^{Leu}$ Adenosine 76–**Cmpd6** adduct was modelled by using the structure of the *E. coli* LeuRS–$tRNA^{Leu}$–**Cmpd9** complex as a template.

Table S1. ***In vitro*** **activities of compounds against** ***Wolbachia*** **LeuRS and efficacy in a** ***Wolbachian*** **infection model.**

Compound ID	Structure	*Wb* LeuRS IC_{50} (μM) *	T ½ (min)	Efficacy in Infected Host EC_{50} (μM) †
**Cmpd1**	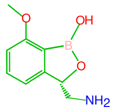	0.34	–	3.77
**Cmpd2**	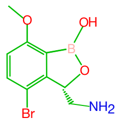	0.40	–	0.36
**Cmpd3**	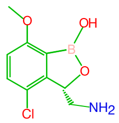	0.29	–	0.33
**Cmpd4**	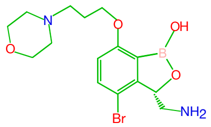	0.21	–	1.43
**Cmpd5**	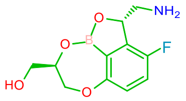	0.34	–	0.56
**Cmpd6**	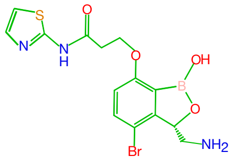	0.06	1.1	>10
**Cmpd7**	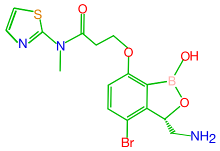	0.14	2.5	6.9
**Cmpd8**	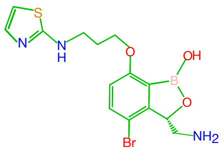	0.12	55	0.57
**Cmpd9**	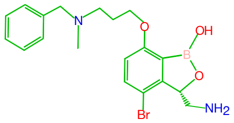	**0.19**	**226**	**0.09**

*The half-maximum inhibitory concentrations (IC_{50}) and half-life inhibition times (T ½) were calculated from aminoacylation assays with recombinant protein and tRNA (see methods). All reactions were performed in triplicate, and the mean values were used to determine an IC_{50} using Prism 4 (GraphPad).

†The half-maximum effective concentrations (EC_{50}) were calculated in cells infected with Wolbachia according to the standard anti-*Wolbachia* (AWOL) screening methods *(18, 27, 29)*. All experiments were done in biological duplicates with duplicate technical triplicates each.

Table S2. Binding energetics of the interactions of compounds and AMP with *Wolbachia* LeuRS determined by Isothermal Titration Calorimetry (ITC).

Compound ID	K_d (nM)	K_a (M^{-1})	ΔG (kcal mol^{-1})	ΔH (kcal mol^{-1})	-TΔS (kcal mol^{-1})
**Nva2AA**	45 000	$2.22\ 10^4$	-5.94	-7.73	1.79
**Cmpd2**	25	$4.01\ 10^7$	-10.36	-7.14	-3.22
**Cmpd6**	44	$2.70\ 10^7$	-10.08	-5.60	-4.48
**Cmpd9**	6	$2.29\ 10^8$	-11.24	-5.93	-5.31

Thermodynamic parameters were calculated from fitting the binding isotherms to a one-site binding site and independent model. Experiments were done at least in duplicates.
All the experiments were performed in the presence of AMP, which acts as a surrogate of ATP or $tRNA^{Leu}$ terminal adenosine (Ade76).

Table S3. Crystallographic data collection and refinement statistics for *Wolbachia* LeuRS and *E. coli* LeuRS crystal structures.

	Wb **LeuRS** ***apo***	***Wb*** **LeuRS + Cmpd6–AMP**	***Wb*** **LeuRS + Cmpd9–AMP**	***Ec*** **LeuRS + Cmpd9–tRNA^{Leu}**
**Data collection**				
Beamline	ESRF ID29	ESRF ID23-1	ESRF ID23-2	ESRF ID30A1
Wavelength (Å)	0.975490	0.979240	0.87313	0.96546
Space group	$P4_3$	$P6_1\ 2\ 2$	$P4_3\ 2_1\ 2$	$P2_1$
Cell dimensions				
a, *b*, *c* (Å)	53.64, 53.64, 267.33	126.63, 126.63, 61.59	52.15, 52.15, 273.56	89.17, 89.17, 90.86
α, β, γ (°)	90.00, 90.00, 90.00	90.00, 90.00, 120.00	90.00, 90.00, 90.00	90.00, 102.55, 90.00
Resolution (Å)*	49.78 – 2.85 (3.10 – 2.85)	109.66 – 1.85 (1.93 – 1.85)	51.22 – 2.14 (2.39 – 2.14)	58.06 – 2.15 (2.37 – 2.15)
R_{sym}			0.22 (2.13)	0.12 (0.98)
R_{meas}	0.05 (2.19)	0.09 (1.89)	0.22 (2.18)	0.13 (1.11)
R_{pim}	-	-	0.04 (0.48)	0.06 (0.50)
$I / \sigma I$	16.6 (1.0)	21.91 (1.62)	12.1 (1.7)	7.1 (1.6)
Completeness (%)	99.9 (99.6)	100.0 (100.0)	93.0 (62.6†)	91.2 (58.4†)
Redundancy	6.8 (6.8)	16.2 (16.8)	29.5 (19.9)	4.5 (4.5)
CC(1/2)	0.99 (0.72)	1.00 (0.67)	0.99 (0.78)	0.96 (0.65)
**Refinement**				
Resolution (Å)	3.0	1.85	2.14	2.15
No. reflections work/free	14906/ 739	24048/ 1281	15508/ 780	44722/ 2224
R_{work} R_{free}	0.2762 0.3263	0.1503 0.1636	0.2117 0.2525	0.2139 0.2652
No. atoms				
Protein	5103	1323	2615	6516
RNA	0	0	0	1795
Ligand	0	46	96	47
Water/Ethylene glycol	11/0	134/4	93/-	377/8
B-factors	146.270	39.653	55.624	53.237
Protein	146.314	38.690	55.649	45.781
RNA	-	-	-	82.425
Ligand	-	24.989	56.085	41.488
Water	125.759	50.360	48.077	44.598

PEG<br>others	-<br>-	39.002<br>98.337	-<br>-	-<br>53.420
R.M.S. deviations				
Bond lengths (Å)	0.002	0.007	0.007	0.007
Bond angles (°)	0.548	1.743	0.91	0.85
Rama Favored (%)	96.49	98.09	97.82	97.90
Rama outliers (%)	0.16	0.00	0.00	0.00

* Data in parenthesis are for the highest resolution shell.

† Reported values are for ellipsoidal completeness calculated by autoPROC STARANISO.
